# Supplementary material for: Imaging through noise with quantum illumination
Source: Sci Adv. 2020 Feb 7;6(6):eaay2652. doi: 10.1126/sciadv.aay2652 (PMC7007263; doi:10.1126/sciadv.aay2652)
Supplement: http://advances.sciencemag.org/cgi/content/full/6/6/eaay2652/DC1 [file supp_6_6_eaay2652__index.html]

Science Advances | Science AdvancesAAASSearchScience AdvancesMenu

## Supplementary Materials

**This PDF file includes:**

- Supplementary Text
- Fig. S1. The quantum illumination advantage as a function of η and *T* plotted with *d* = 0.0016; *pr* = 0.0016; ε = 0.5.
- Fig. S2. The quantum illumination advantage as a function of η and ε (plotted with *d* = 0.0016; *pr* = 0.0016; *T* = 0.0016.
- Fig. S3. Imaging using quantum illumination within an increasing thermal background.
- Fig. S4. Quantum illumination advantage *A* calculated over a range of increasing levels of thermal illumination.
- Fig. S5. Plot of the quantum illumination advantage *A* for the system under differing levels of optical loss.
- Fig. S6. The bit error rate *P*err of detecting a target calculated over a range of thermal light levels.
- Fig. S7. The bit error rate *P*err of detecting a target calculated over a range of thermal light levels using the second method.
- Table S1. Table of the average s weight values calculated over a range of thermal light levels using the second method for each of the different levels of thermal illumination.

Download PDF

**Files in this Data Supplement:**

- Adobe PDF - aay2652\_SM.pdf
