# Supplementary material for: Imaging through noise with quantum illumination
Source: Sci Adv. 2020 Feb 7;6(6):eaay2652. doi: 10.1126/sciadv.aay2652 (PMC7007263; doi:10.1126/sciadv.aay2652)
Supplement: Download PDF [file aay2652_SM.pdf]

Science Advances
AAAS

advances.sciencemag.org/cgi/content/full/6/6/eaay2652/DC1

Supplementary Materials for

Imaging through noise with quantum illumination

T. Gregory, P.-A. Moreau, E. Toninelli, M. J. Padgett*

*Corresponding author. Email: miles.padgett@glasgow.ac.uk

Published 7 February 2020, *Sci. Adv.* **6**, eaay2652 (2020)
DOI: 10.1126/sciadv.aay2652

This PDF file includes:

Supplementary Text
Fig. S1. The quantum illumination advantage as a function of η and T plotted with $d = 0.0016$; $p_r = 0.0016$; $\varepsilon = 0.5$.
Fig. S2. The quantum illumination advantage as a function of η and ε (plotted with $d = 0.0016$; $p_r = 0.0016$; $T = 0.0016$.
Fig. S3. Imaging using quantum illumination within an increasing thermal background.
Fig. S4. Quantum illumination advantage A calculated over a range of increasing levels of thermal illumination.
Fig. S5. Plot of the quantum illumination advantage A for the system under differing levels of optical loss.
Fig. S6. The bit error rate P_{err} of detecting a target calculated over a range of thermal light levels.
Fig. S7. The bit error rate P_{err} of detecting a target calculated over a range of thermal light levels using the second method.
Table S1. Table of the average s weight values calculated over a range of thermal light levels using the second method for each of the different levels of thermal illumination.

Supplementary Text

Light Level

The regime in which is used as a baseline when no additional environmental losses or thermal light are introduced is the regime defined by Tasca et al. (2013) (28) in which the thresholded events per pixel per frame from the detection of SPDC events matches the thresholded event rate due to the clock induced charge of the camera. The clock induced charge of the camera using the aforementioned settings is ~0.0016 events per pixel per frame and therefore the event rate for the regions within the SPDC beams is set to be ~ 0.0032 events per pixel per frame. For this system the AND-efficiency, given as η below, has been estimated to be 0.0021056. This value is calculated to be the proportion of events that occur in the reference beam that have an anticorrelated event in the probe beam corrected for randomly correlated events. This value is determined from the data used to generate the correlation peak as displayed in Fig. 3 in the main text. The quantum efficiency of the EMCCD camera (Andor iXon ULTRA 888 DU-888U3-CS0-#BV) is quoted as 77.75% when cooled to -100°C while we operate the camera at -90°C. However, as we describe above, we threshold each frame and this further reduces the detection efficiency as not all photons detected by the camera are registered as events in the thresholded frames.

Theory on contrast enhancement

We establish here an expression for the expected quantum illumination advantage as a function of experimental parameters. We show how the advantage depends on the losses in the target or probed arm, and as a function of the amount of thermal light. We note p_r the rate of SPDC photons detected in the reference arm, η is the apparatus arm efficiency i.e. including losses occurring in the quantum illumination arm at the exclusion of target losses such that the efficiency of the reference arm from the crystal to the camera included is $\eta_r = \eta$ and of the probe arm from the crystal to the camera included is $\eta_p = \eta\varepsilon$, where ε is the probe arm efficiency. This gives a detection rate of SPDC photons in the probe arm of $p_p = \varepsilon p_r$. We note d the dark count rates and T the thermal light rate. One can then write the AND event rate with the quantum correlated light as follows

$$R^Q_{Bright} = \varepsilon\eta p_r + (p_r(1-\varepsilon\eta)+d)(\varepsilon p_r(1-\eta)+d+T) \tag{S1}$$

$$R^Q_{Dark} = (p_r + d)(d+T)$$

Where R^Q_{Bright} is the AND event rate detected in the bright part of the object and R^Q_{Dark} is the AND event rate detected in the dark parts of the object. One can as well express the rates in the classical simple average as follows

$$R^C_{Bright} = \varepsilon p_r + T + d \tag{S2}$$

$$R^C_{Dark} = T + d$$

From these equations one can express the quantum illumination advantage A as a function of the experimental parameters by using

$$A = \frac{(R^Q_{Bright} - R^Q_{Dark})(R^C_{Bright} + R^C_{Dark})}{(R^Q_{Dark} + R^Q_{Bright})(R^C_{Bright} - R^C_{Dark})} \quad \text{(S3)}$$

We have plotted on following figures the contrast advantage A as a function of η and T (plotted with $d = 0.0016$; $p_r = 0.0016$; $\varepsilon = 0.5$) in fig. S1, and as a function of η and ε (plotted with $d = 0.0016$; $p_r = 0.0016$; $T = 0.0016$) in fig. S2. One can observe that the advantage not only increases with increasing T but also when losses are added i.e. when ε decreases. Note however that adding too bright thermal light T will result in the saturation of a single photon pixel detector and therefore result in the failure of the protocol and the loss of any advantage. This technical issue can however be solved by using a detector array such as a SPAD array with a higher temporal resolution therefore reducing the likelihood of it being saturated by the thermal light.

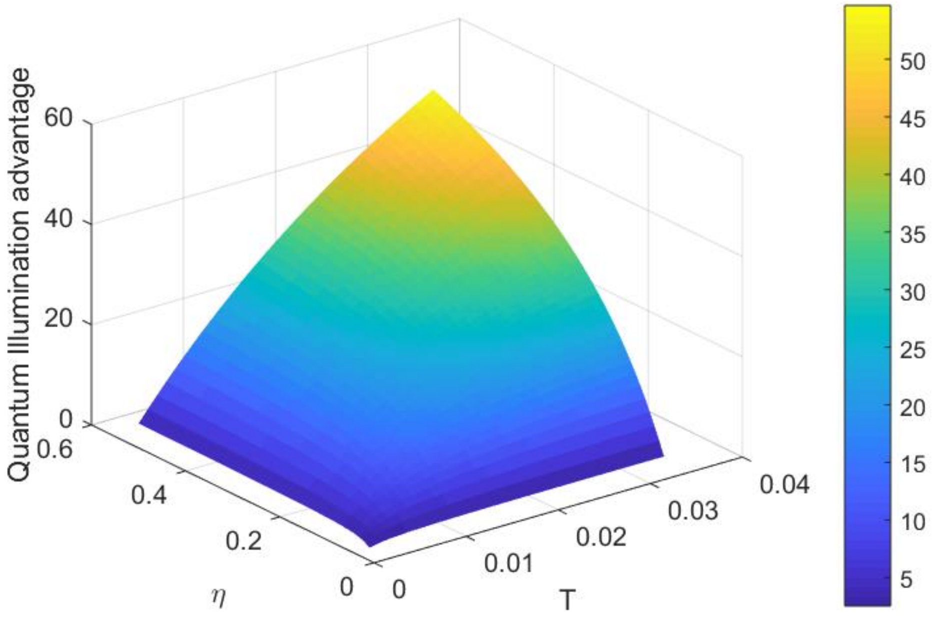

Fig. S1. The quantum illumination advantage as a function of η and *T* plotted with *d* = 0.0016; p_r = 0.0016; ε = 0.5.

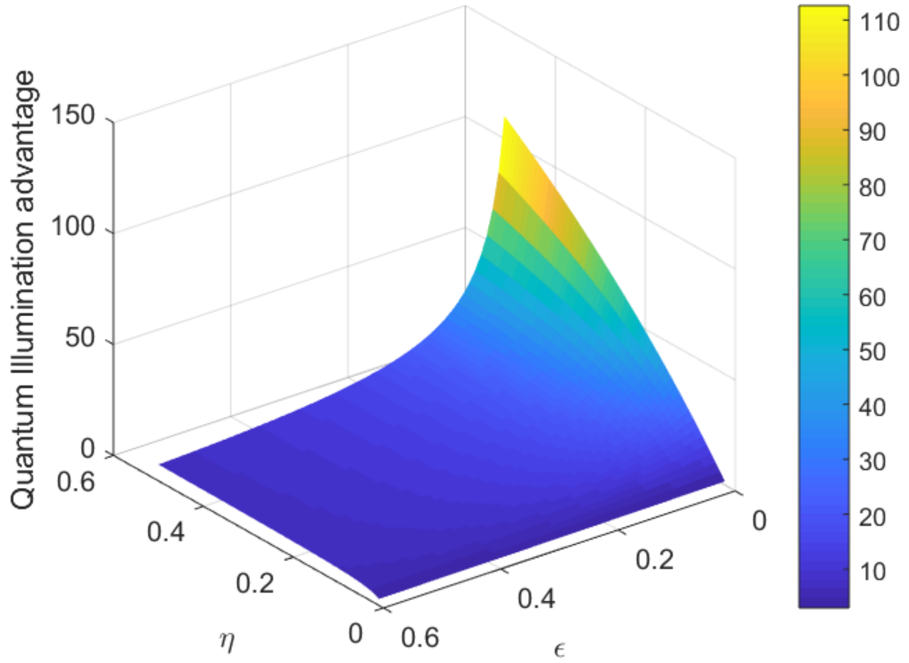

Fig. S2. The quantum illumination advantage as a function of η and ε (plotted with *d* = 0.0016; p_r = 0.0016; *T* = 0.0016.

<u>Quantum illumination advantage in the presence of thermal noise</u>

Figure S3 compares the results under increasing thermal light levels. It is seen that despite the introduction of increasing environmental noise events into the probe arm, the quantum illumination advantage, A, increases as the level of thermal illumination increases. This trend may also be seen in the plot of the quantum illumination advantage, A, plotted against the thermal illumination level in fig. S4. This trend is the expected behaviour from the equations we presented in the preceding section.

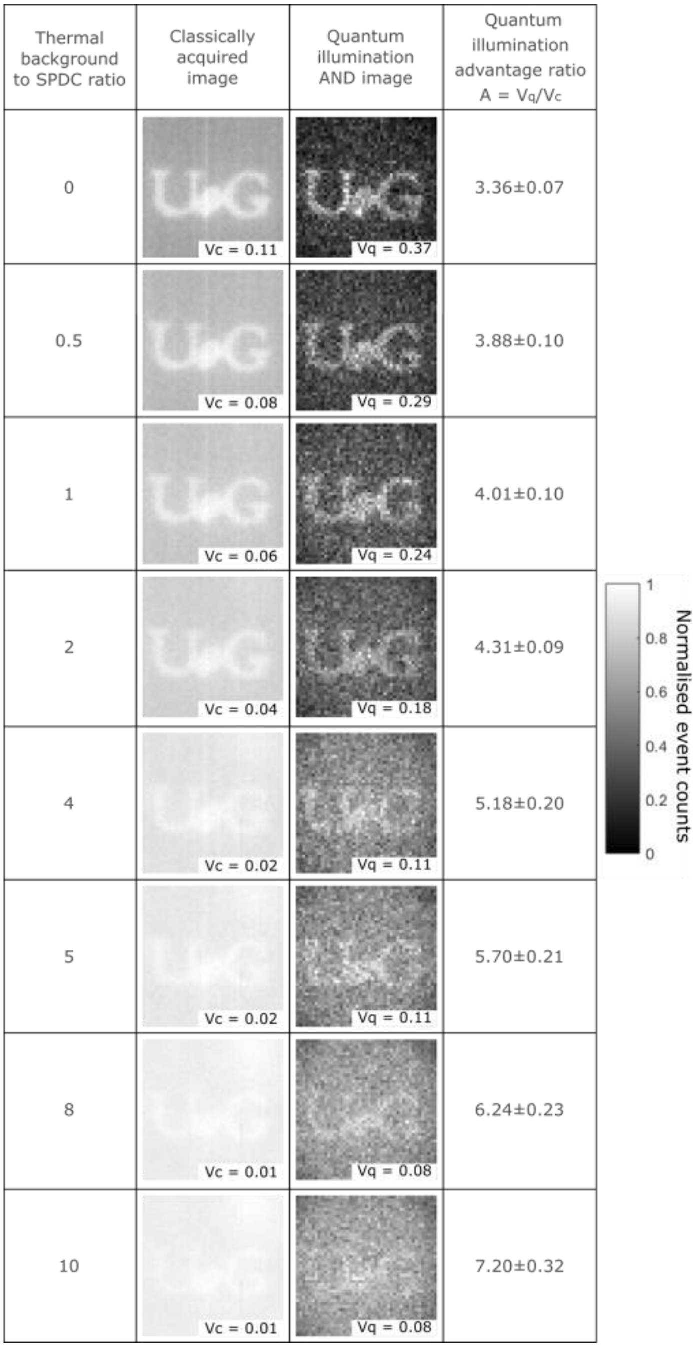

Fig. S3. Imaging using quantum illumination within an increasing thermal background. Images of the UoG object with the classical image created by averaging all frames (second column), and the quantum illumination AND-image built from the sum of results of performing an AND-operation to select correlated events in the reference and probe beams (third column). The UoG object illuminated by the probe beam is imaged under the conditions of an increasing thermal background level (see the ratio of thermal illumination to SPDC illumination at 710 ± 5 nm on the left). The quantum illumination advantage A under these thermal illumination levels for images constructed over 1.5 million frames is displayed on the right. The given uncertainty is the standard error on the mean calculated using blocks of 100,000 frames. Images are 45 x 45 pixels.

Figure S4 shows the quantum illumination advantage A under increasing levels of thermal noise introduced into the system. Points additional to those for which results are shown in fig. S3 are included.

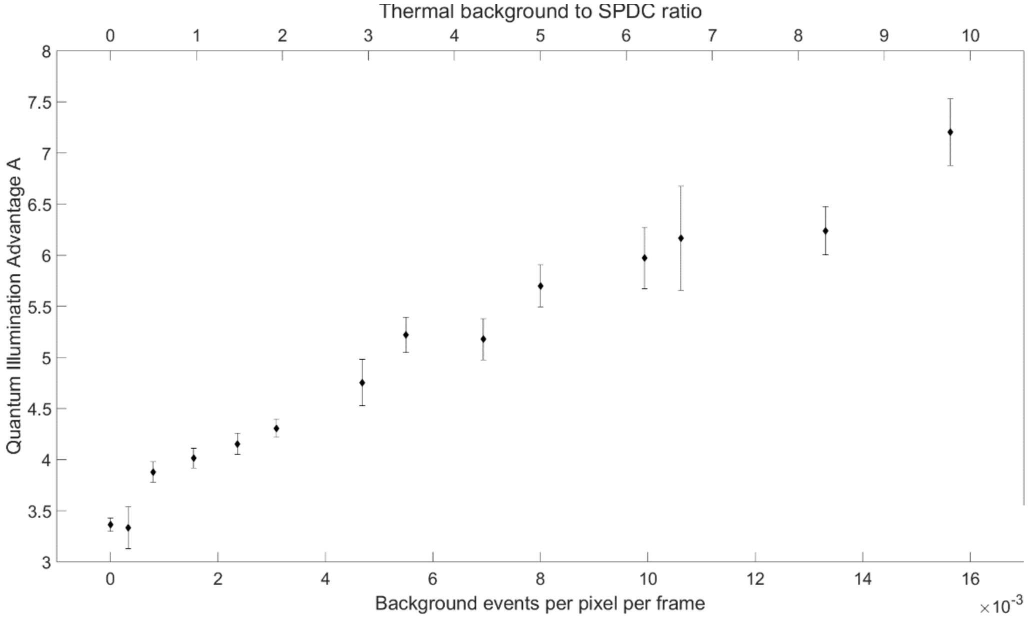

Fig. S4. Quantum illumination advantage *A* calculated over a range of increasing levels of thermal illumination. The ratio of thermal background to SPDC illumination is given for the range of levels of thermal illumination. The given uncertainty is the standard error on the mean calculated using blocks of 100,000 frames.

<u>Quantum illumination advantage in the presence of losses</u>

Figure S5 shows the quantum illumination advantage A under identical thermal light levels ~0.0016 thermal events per pixel per frame with additional optical losses introduced into the probe beam after interaction with the object. It is seen that despite the introduction of losses into the probe arm, the quantum illumination advantage, A, increases as the level of losses increases as may also be seen in Fig. 5 in the main text. This trend is the expected behaviour from the equations we presented in the preceding section.

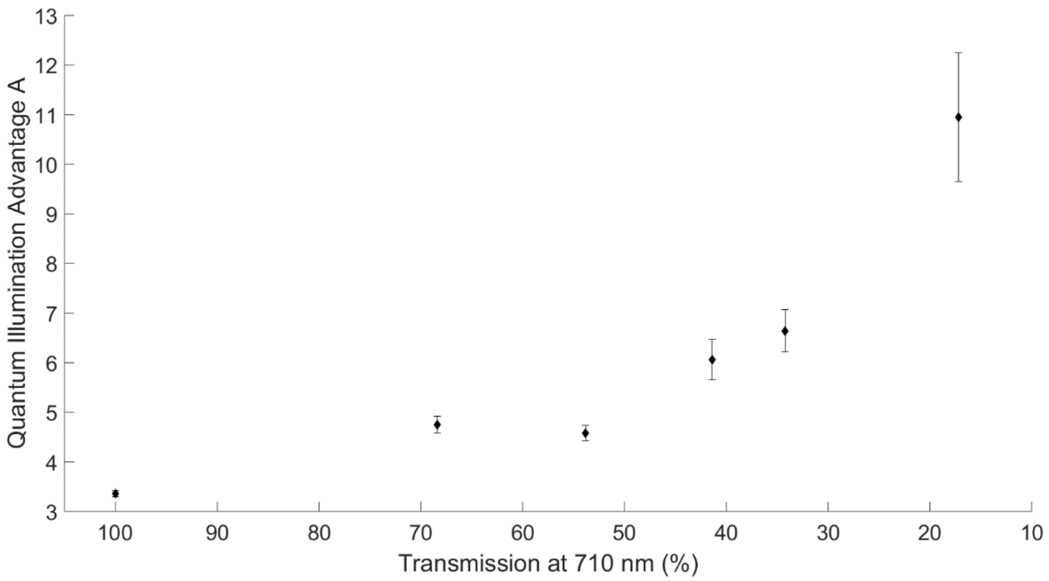

Fig. S5. Plot of the quantum illumination advantage *A* for the system under differing levels of optical loss. The quantum illumination advantage A is assessed under these levels of optical losses for images constructed over 1.5 million frames. The given uncertainty is the standard error on the mean calculated using blocks of 100,000 frames.

Bit Error Rate determination method and analysis

In contrast to the theoretical considerations reported in (1,2), that assume the quantum illumination apparatus is ideal in that the idler arm efficiency is unity, we cannot make such an assumption. As a consequence we have to distinguish two strategies in the way one can guess the presence of a target. The first one where we do not use any knowledge about the noise or the target and for which the use of the AND-image alone will surpass a classical strategy. The second where the system is assumed to be fully calibrated and in which we know the noise levels and the transmittance of the target and for which one needs to use both the correlated AND-image data and the classical image data acquired through the quantum illumination protocol to obtain an advantage. We show here in both cases that observing a contrast advantage in the AND-images implies that one would have also an advantage in guessing the presence or absence of the target.

1) With a blind strategy

We describe here the method used to determine the bit error rate in estimating the presence or absence of a target, through both a quantum illumination and a classical illumination protocol.

The bit error rate P_{err} is determined both by the rate of predicting the presence of the target when it is absent $P_{Dark}(1)$ and the rate of prediction of its absence when it is present $P_{Bright}(0)$.

This can be written under the hypothesis of equal probability to have the target present or absent

$$P_{err} = \frac{1}{2} P_{Dark}(1) + \frac{1}{2} P_{Bright}(0) \quad \text{(S4)}$$

To determine the error rate experimentally, one simply recognises the detection of zero events on a pixel of the image that comprises the target as revealing the absence of the target object, and the detection of one event or more as revealing the presence of the target object and try to find the optimal classical strategy in doing so (1). Knowing the ground truth (i.e. the target shape) one can assess the error rate in determining the presence or absence of the target object through this method.

In order to find the optimal classical strategy and show that the quantum strategy exhibits an advantage we make the classical prediction on an image that is composed of the sum N thresholded frames and search the value of N that minimises the classical error rate P_{err}^{C} for our particular experimental conditions and when no assumption of the object or the noises present can be made. In the case of quantum illumination the image is the sum of the result of the AND-operation between the reference and probe beams and therefore a signal event is added to the resulting AND-image only when a detection event occurs on the correlated idler pixel, thus post selecting the prediction. For fair comparison we use and sum the same number of post selected events in the images used for quantum illumination as were present in the sum of N thresholded frames. Which means that because the fill factor of the idler events in the detected reference beam is given by $(p+d)$ we use $\frac{N}{(p+d)}$ frames to compose an image using the sum of post-selected events only, and make our prediction based on these images. From knowing the ground truth one can again evaluate the bit error rate.

It is important to note that because N is optimised for the classical scheme only, the quantum illumination protocol may be sub-optimal under certain conditions, nevertheless we find a systematic advantage.

Practically P_{err} is evaluated by applying a mask of the UoG target object and finding the number of background pixels that feature a detection event (false positive) and the number of pixels that comprise the UoG object but have no detected events (false negative). From our data the bit error rate across a range of thermal light levels was determined

One can predict the theoretical values of the error rate in the classical P_{err}^{C} and in the quantum illumination P_{err}^{Q} cases. Starting first with the classical case, under the hypothesis of equal probability to have the target present or absent and when one makes the prediction on an image composed by the sum of N frames one can write

$$P_{err}^{C} = \frac{1}{2}(1-(P_{Dark}^{C}(0))^{N}) + \frac{1}{2} P_{Bright}^{C}(0)^{N} \quad \text{(S5)}$$

Where $P_{Dark}^{C}(0)=1-(d+T)$ is the probability to detect no photons in a single frame when the target is absent within a particular pixel. And $P_{Bright}^{C}(0)=1-(\varepsilon p+d+T)$ is the probability to detect no photons in a single frame when the target is present within a particular pixel.

The quantum illumination strategy consists of guessing the presence or absence of the target, based on post selected detection i.e. by counting the probe events or non-events happening at a particular pixel only when a reference event is detected on the corresponding correlated pixel. When making the prediction of presence or absence on images composed of N added such post-selected events per pixel one can write

$$P_{err}^{Q}=\frac{1}{2}(1-(P_{Dark}^{Q}(0))^{N})+\frac{1}{2}P_{Bright}^{Q}(0)^{N} \qquad \text{(S6)}$$

Where $P_{Dark}^{Q}(0)=1-(d+T)$ is the probability to detect no photons within a particular pixel of a single frame when an idler photon has been detected on the correlated pixel position and when the target is absent. And $P_{Bright}^{Q}(0)=\frac{p}{p+d}(1-(\varepsilon\eta+T+d))+\frac{d}{p+d}(1-(\varepsilon p+T+d))$ is the probability to detect no photons within a particular pixel of a single frame when an idler photon has been detected on the correlated pixel position and when the target is absent.

We used these equations (S5) and (S6) to fit the experimental data reported in fig. S6.

Importantly one can note that the advantage in estimating the presence or absence of the target is based on the same mechanism as for the advantage obtained in contrast. It can be understood by realising that for equal values of accumulation of N events used in the quantum illumination and the classical case one would obtain the same background in case of the absence of the target and therefore the same error rate in wrongly predicting the absence of the target (the two first terms in equations (S5) and (S6) are equal). This means that the quantum illumination advantage in determining the presence or absence of the object in such conditions is based on a higher number of detected events in the bright parts of the images compared to the classical images. This higher rate of detected events means that the guessing the presence of the object is more accurate with quantum illumination. Therefore when the quantum and classical images have the same background, it is the higher intensity of the bright parts of the quantum image that explains the increased prediction accuracy, an increased intensity for the pixels in the frame that comprise the object with an equivalent background means also that the quantum image will exhibit a higher contrast than the classical image.

2) When the target transmittance and noises are known
Following (2) one could try to make predictions on the presence or absence of the object when the transmission of the target ε and the noise levels are known. In such a case the

best strategy is to accumulate events and use a threshold level that minimises the error rate. This can be found theoretically by knowing the system parameters or by calibrating the system with such parameters.

In such a context and in contrast with the theoretical considerations reported in (2) that assume that the idler arm efficiency is ideal, we cannot make such an assumption. A consequence is that if in our case we simply use the AND-images to perform our estimations of the presence of the target, the results will be worse than with the classical image, because our classical image contains a greater number of events and therefore less shot noise than the quantum illumination AND-image.

However with the complete set of data acquired through quantum illumination, one can still improve the bit error rate compared to the classical case. To do so, one needs to use a combination of both the classical data (classical images) and the non-classical data (the AND-images) in a similar way that the optimal strategy for sub-shot noise measurement is to use both correlated and uncorrelated data (20). To understand that one can observe a quantum illumination advantage means that the ensemble of post selected events detected within the quantum illumination AND-image image are more valuable than the same number of non-correlated events in predicting the presence of the object. Indeed for an equal number of events the noise in both the bright parts and the dark parts of the image will be the same in both the AND-image and a classical image, however the contrast is higher in the AND-image than classical image with the same number of events. This is because a higher contrast means that the places where the object is present and absent are further apart in intensities, and therefore for same shot noise level the optimal guess will be more accurate in the AND-image. However because of the non-ideal idler arm efficiency η one will have more events in the total classical image than in the AND-image. The best strategy then is to use a combination of both images in order to give more weight to the correlated events than to the uncorrelated ones. And as long as the AND-image exhibits a contrast advantage, this will lead to an improvement of the bit error rate over the simple use of the classically acquired image.

Practically this means that one would have to use an optimal image

$$I_{Opt} = (1-s)I_{Average} + sI_{AND} \tag{S7}$$

Where the weight s ($0 \leq s \leq 1$) is in particular dependent on the value of η , the greater the efficiency η the more events I_{AND} are kept and the greater s should be, $s=1$ if $\eta=1$. It also depends on any other parameters affecting the contrast advantage. The higher the contrast advantage, the more useful I_{AND} will be and therefore the greater s should be. In particular a greater timing resolution in the detection of the correlations can further reduce the probability of detecting false coincidences due to the thermal light at a given light level. This means that a better timing resolution such as those accessible with SPAD arrays can improve the contrast advantage and will therefore mean that the value used for s in such circumstances should be greater.

Finally, we would like to conclude this paragraph by remarking again that a contrast advantage in I_{AND} implies that it can be used to build a strategy that is better than the classical strategy, which means that a contrast advantage through quantum illumination implies an error rate advantage.

Bit error Rate Results

Here we present an analysis of how this protocol may be used in an application where the presence or absence of an object needs to be assessed. This is the context in which Lloyd (1) originally proposed the quantum illumination protocol and in which it has clear applications in realising quantum LIDAR or quantum radar applications. The error rate in detecting the presence or absence of an object in the probe beam path is assessed over a range of light levels using the 'blind' strategy in which no prior information is assumed in fig. S6. The advantage in the probability of successfully determining the presence or absence of an object for quantum illumination may be seen over the range of thermal light levels. The points lie below the curve for the quantum illumination AND-image due to the thermal illumination not being entirely flat as may be seen in the images presented in fig. S3.

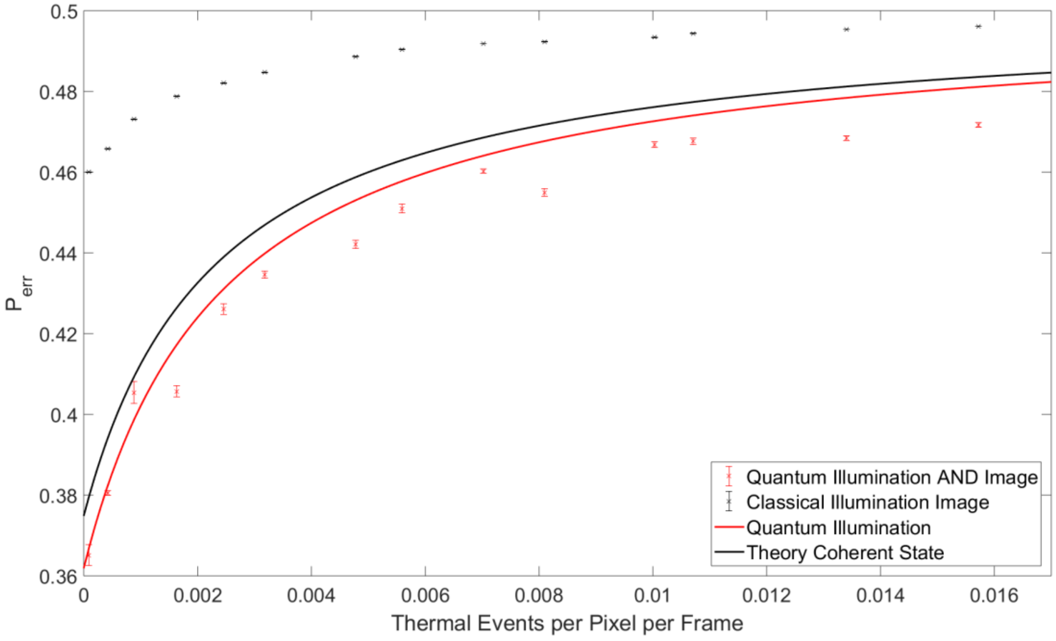

Fig. S6. The bit error rate P_{err} of detecting a target calculated over a range of thermal light levels. The classical data is represented by the black crosses and the quantum illumination AND-image by the red crosses. The curve in black represents the theoretical optimum bit error rate for an image constructed from coherent state illumination. The red curve represents the equivalent curve for the quantum illumination AND-image calculated using experimental parameters. These theoretical curves are valid under the assumption of an unknown background and target object and assuming Poissonian camera dark noise and thermal light. Error bars are the standard error on the mean for the bit error rate.

In the case of the second strategy a weighted sum (see equation S7) of the classical image and the quantum illumination AND-image is used to calculate the bit error rate. For both the classical image and also the weighted sum of the classical and AND-image the mean and standard deviation of both the background pixels (μ_{bg} and σ_{bg}) and also of the pixels that comprise the UoG object (μ_{UoG} and σ_{UoG}) are found. The threshold T is then set such that

$$T = \mu_{UoG} - n_{sd}\sigma_{UoG} = \mu_{bg} - n_{sd}\sigma_{bg} \quad \text{(S8)}$$

where

$$n_{sd} = \frac{\mu_{UoG} - \mu_{bg}}{\sigma_{UoG} + \sigma_{bg}} \quad \text{(S9)}$$

The images are then thresholded appropriately and the bit error rate calculated. This is performed over a range of weights s (0 ≤ s ≤ 1) and the minimum value for the bit error rate determined.

It may be seen in fig. S7 that the advantage in the bit error rate is decreased compared to that in the 'blind' strategy. This is due to non-ideal idler arm efficiency resulting in a non-unity value for the weight value s.

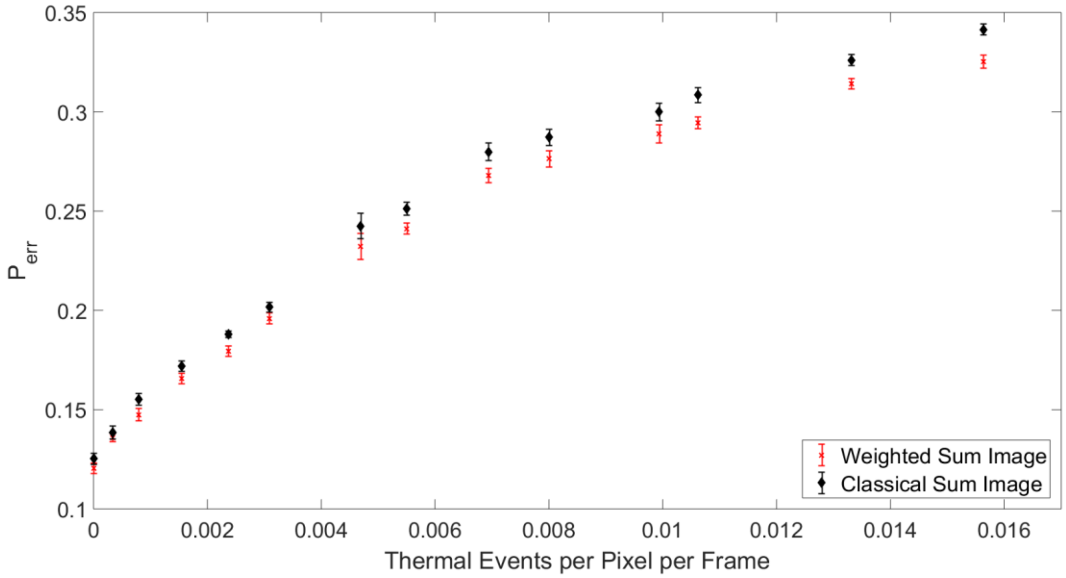

Fig. S7. The bit error rate P_{err} of detecting a target calculated over a range of thermal light levels using the second method. The purely classical data is represented by the black crosses and the weighted sum of the classical image and the quantum illumination AND-image by the red crosses. The given uncertainty is the standard error on the mean calculated using blocks of 100,000 frames.

The values of s calculated to optimise bit error rate of the compound images as per method 2 of the bit error rate calculation for the UoG objects under a range of differing thermal illumination conditions are shown in table S1 accompanying fig. S7. The average value of s for this set of data is 0.7702 +/- 0.0401, and the optimal values found for each light level seems consistent (within the error bars). The fact that these values for s are non-unity indicates that a greater advantage may be achieved should the system efficiency be further increased, but also with a better correlation timing resolutions that could lead to an improved contrast advantage.

Table S1. Table of the average *s* weight values calculated over a range of thermal light levels using the second method for each of the different levels of thermal illumination. The given uncertainty is the standard error on the mean calculated using blocks of 100,000 frames.

Background events per pixel per frame ($x10^{-4}$)	s weight value
0	0.6542 +/- 0.0544
4	0.7642 +/- 0.0687
8	0.7678 +/- 0.0255
16	0.7457 +/- 0.0419
24	0.7785 +/- 0.0110
32	0.6610 +/- 0.0638
48	0.8389 +/- 0.0096
55	0.7481 +/- 0.0570
70	0.8118 +/- 0.0180
80	0.8160 +/- 0.0204
100	0.8021 +/- 0.0284
104	0.7991 +/- 0.0066
128	0.8166 +/- 0.0215
160	0.7789 +/- 0.0536
